# What do times of crisis reveal about the ‘Total’ nature of prisons? Analysing the impacts of the COVID-19 crisis within the Scottish prison system

**DOI:** 10.1177/26338076231165116

**Published:** 2023-03-28

**Authors:** Matthew Maycock

**Affiliations:** 2541 Monash University, Melbourne, Australia

**Keywords:** Prison, Goffman, the ‘total instutition’, Covid-19 in prison, crisis within prison

## Abstract

Times of crisis within prison settings either at a system-wide level during times of riots or during pandemics or at more personal levels during time in segregation can be particularly challenging times when the prison can feel more ‘total’ than other times. Goffman's influential work outlines a particular interpretation of the parameters of the total institution, of which prisons were one manifestation. In the years following its publication, a wide range of research has sought to subvert the notion that prisons are total institutions, suggesting a greater permeability of contemporary prison walls. This article calls for a re-consideration of this dismissal, and a reconnection and critical engagement with Goffman's original parameters within the context of the COVID-19 pandemic and associated lockdown. The lockdown in response to COVID-19 in prison settings has resulted in many prison jurisdictions rolling back many of the erosion of the prison looking and feeling like a total institution. Through the analysis of 19 letters received from 8 people in custody in one Scottish prison, there emerges a reframed and reconsidered permeability of prison walls. For the participants in this study, the experiences of the COVID-19 lockdown complicate much of the recent critique of the relevance of the total institution as a theoretical frame to analyse contemporary prisons. This article argues that by considering the ways in which the COVID-19 pandemic, it is possible to observe a more essential quality of contemporary imprisonment, obscured through decades of penal reform but one that emerges during times of crisis.

## Introduction

Prison systems have been described as systems in ‘crisis’ (Evans, 1980; Fitzgerald & Sim, 1980; Toch, 2007), in some instances, this has been in reference to the crisis of overcrowding (Cox & Rhodes, 1990; Losel, 2007; Sessar, 1994). The prison crisis literature has explored aspects of ‘crisis’ at multiple levels within prison settings, either personally through prisoners spending time in segregation or in mental health crisis, as well as system-wide form of crisis, for example, during riots, outbreaks of infectious diseases, etc. A common thread emerging across these studies is that times of crisis at these different levels are often associated with increasingly restrictive and/or closed-off conditions within prison. At an individual level, when people are moved to the segregation units or protection wings of prisons, the prison regime is far more constrained and cut off (Brown, 2020; Shalev & Edgar, 2015). There has also been analysis of responses to riots and other forms of disorder in prisons are considered as times of crisis, that similarly are associated with greater restrictions on the prison regime (Adams, 2016; Carrabine, 2005; Colvin, 1992).

Through the analysis of the impacts of COVID-19 in the Scottish prison system, this article provides new insights into the implications of times of crisis within prison settings, and what times of crisis at other times in prison tell us about the nature of contemporary imprisonment.

Within the particular context of the COVID-19 pandemic and associated crisis within prison settings, this article extends the above literature. Times of ‘crisis’ and what this justified in relation to more a punitive and restrictive prison regime, is explored through the analysis of the impacts of COVID-19 pandemic within the Scottish prison system. The response to the crisis that the COVID-19 pandemic represented within prison settings by governments and prison administrators overnight has eroded the many years of more progressive reforms within prison systems, that had brought prisons and the communities around them closer. Therefore, this article argues that times of crisis such as the COVID-19 pandemic reveal a more fundamental and ‘total’ quality of prison settings.

This article uses Erving Goffman's theory of the *total institution,* originally outlined in his book Asylums (1961), a theory that remains widely debated and contested within contemporary penology (Davies, 1989; Farrington, 1992; Mac Suibhne, 2011; Moran, 2013, 2014; Schliehe, 2016). In recent years, this debate has tended to suggest a declining relevance of the theory, emphasising an increasing porousness and permeability of prison walls (Baumer et al., 2009; Ellis, 2021; Moran, 2013), with the extent to which prisons are discrete spaces that are distinct and separate from community contexts brought into question. This article re-examines Goffman's theory of the total institution in light of the impacts of COVID-19 in prison settings, and critically engages with the influence that prison administrators have in terms of the extent to which prisons are porous and connected to communities, or not. This is of critical concern in terms of the analysis of the experience of imprisonment, given that as Sykes indicated, one of the main deprivations or frustrations of imprisonment relates to ‘isolation from the free community’ (Sykes, 1958, p. 79). This article argues times of crisis such as the COVID-19 pandemic,

Across diverse areas of analysis, this literature implies that prisons have an increasing resonance or synergy with community contexts, with a closer and an increasing number of connections between custody and community (Comfort, 2009). This article critically engages with this trend within the devolved Scottish penal context (Croall et al., 2016; Morrison, 2016; Morrison, 2012; Sparks & Morrison, 2015) where a range of recent penal policies has sought to locate people in custody closer to their communities. Policy development in relation to Scottish prisons emerges as exhibiting an ambition to increase the equivalence of services within custody and the community. For example, in 2011, healthcare provision in Scottish prisons was transferred from the Scottish Prison Service (SPS) to NHS Scotland delivery, in order to increase the equivalence and continuity of healthcare provision in custody and the community (RCN, 2016). This article calls for a reconsideration of the critique of Goffman's theory and the trends identified in Scottish prison policy, through the analysis of 19 letters received from 8 participants located in one long-term adult male prison in Scotland. This article argues that this trend towards blurring the boundaries between prison and community has been compromised and/or gone backwards as a result of COVID-19. Additionally, this article argues that times of crisis such as the COVID-19 pandemic help to illustrate a more fundamental ‘total’ quality of prisons, which is most evident during times of crisis.

Due to restrictions on face-to-face data collection, this study used a correspondence methodology to gain insights into everyday life in prison during the COVID-19 lockdown (Maycock, 2021a, 2021b; Maycock & Dickson, 2021). This study analyses the letters received from eight adult male participants all serving long-term sentences^1^, in order to explore the experiences of COVID-19 within one Scottish prison (Maycock, 2021a; Maycock & Dickson, 2021). The findings of this study complicate the official discourse relating to COVID-19^2^, in so far as the COVID-19 lockdown in prison emerges as a hardening or deepening of the experience of prison (Maycock, 2021a), with this time emerging as particularly challenging for people in custody as well as prison staff (author, forthcoming). This article illustrates that the participants in this study were reconsidering and rethinking what being in prison meant to them as a consequence of the implications of COVID-19 pandemic and lockdown on everyday life in prison. In some respects, this article analyses a growing distance between participants, their family, friends and wider communities during the COVID-19 lockdown (increasingly cut off). The article also analyses a number of contrasting experiences and processes as a consequence of the participants in the study reflecting on their experiences of the COVID-19 lockdown which also suggests a greater closeness to aspects of the COVID-19 pandemic. The greater closeness to the community (increasingly close) is framed around the perceived threat of viral transmission from the community as well as the availability of COVID-19 vaccines.

This article argues that these contradictory processes of both increasing distance as well as closeness between life in custody and community more widely facilitate a re-examination of the relevance of Goffman's theory within the context of the COVID-19 pandemic. The article concludes with a discussion that situates this article's analysis in a wider context of studies of other aspects of crisis within prison settings, and what the response to crisis illustrates in relation to contemporary imprisonment. Through considering the implications of times of crisis within prison settings and what this means for these spaces in terms of their total qualities. The ways in which the COVID-19 pandemic was experienced and described as a ‘crisis’ is returned to throughout this article, as this is something that significantly influences official narratives relating to the COVID-19 pandemic in prison. This is a narrative that has justified a particularly punitive and isolating regime with most prisoners confined to their cells for extended periods of time (Maycock, 2021a; Maycock & Dickson, 2021). Within this context, the COVID-19 pandemic has been called the biggest public health crisis of our time (Scottish Government, 2020a), with the particular implications of this crisis within prison settings forming the focus of this article and wider study. Ultimately, this study suggests that times of *crisis,* either personal or systematic within prison settings, facilitate insights into the nature of contemporary imprisonment that point to a more essential and *total* quality of prisons that can have a profound impact on everyday life in prison.

## Goffman and the prison as a total institution

Goffman's book Asylums (1961) is a collection of four essays that have had a wide impact across a range of disciplines, including penology. The term total institution developed in this book, has a wide range of definitions and meanings, however, for Goffman, a ‘total institution’ is defined as:

…a place of residence and work where a large number of like-situated individuals, cut off from the wider society for an appreciable period of time, together lead an enclosed, formally administered round of life. (Goffman, 1961: xiii)

The important part of this definition for this article relates to the nature and extent of being *cut off*, or *enclosed*, something that has been the focus of subsequent analysis and critique. It is in the particular context of the COVID-19 pandemic and lockdown in prison that this article reconsiders the ways in which people in prison were both *cut off* in new ways reconnecting with Goffman. Importantly, there emerge some counter processes where participants felt *connected* in new ways to communities outside of prison through, for example, the availability of COVID-19 vaccines. A tension emerges between these processes, where the things participants were cut off from (families, peers, educational opportunities, information, etc.) were felt as additional to or the deepening of the pains of imprisonment (Sykes, 1958). Conversely, the things that participants felt in some ways closer to (the COVID-19 virus, lockdown measures and COVID-19 vaccinations) were also considered negatively. The narrative around the COVID-19 virus in particular emerges for the participants in this study as a threat imported by prison staff into the prison from the wider community. The intertwined processes of being *cut off* alongside being *closer* to life and events outside of prison as a consequence of the COVID-19 pandemic and lockdown constitutes a new context to re-examine the relevance of the total institution as an analytical lens through which to analyse the contemporary experience of imprisonment. Goffman indicates that institutional settings are diversely configured on a continuum from closed to open, with those completely closed considered ‘total’:

Their encompassing or total character is symbolized by the barrier to social intercourse with the outside and to departure that is often built right into the physical plant, such as locked doors, high walls, barbed wire, cliffs, water, forests, or moors. (1961, II)

Once more in the quote above, the ways in which total institutions are separated from other settings are emphasised, the barrier being both symbolic and real. The notion of institutional ‘outside’ and ‘inside’ as well as the literal and theoretical construct of the wall enclosing closed or total institutions has been the focus of significant subsequent debate within penology. These are long-standing questions within penology that this article argues has new answers shaped by the COVID-19 pandemic and lockdown. According to Goffman, there were five ‘rough groupings’ of total institution that included jails and penitentiaries (ibid, 4–5):

“A third type of total institution is organized to protect the community against what are felt to be intentional dangers to it, with the welfare or the persons thus sequestered not the immediate issue; jails, penitentiaries, P.O.W camps and concentration camps.” (Goffman, 1961, p. 5)

A particular feature of this type of total institution, in contrast to the others analysed by Goffman relates to the way in which people are sent to prison involuntarily (1961, p. 118). The encompassing or ‘total’ nature of these institutions is characterised both by a harshness of conditions within them, alongside the social disconnections that are a consequence of being within them:

However harsh the conditions of life in total institutions, harshness alone cannot account for this sense of life wasted; we must rather look to the social disconnections caused by entrance. (1961, p. 77)

Goffman's work on total institutions is still widely debated and used to analyse and discuss contemporary prisons (Ellis, 2021). This article further contributes to this long-standing debate, adding a new perspective developed out of the analysis of the impacts of COVID-19 in prison settings.

### Critiques of Goffman's total institution and the increased ‘porousness’ of prisons

Alongside the widespread application of the theory of the total institution to prisons and other intuitions, a range of critiques relating to aspects of the ‘porous’ nature of prisons and prison walls or barriers have been developed (Davies, 1989), in particular in the field of carceral geography (Moran, 2015). In relation to the permeability of prison spaces, Moran (2012, 2014, 2015) has a long-standing critical engagement with Goffman's total institution applied to prison settings, including, for example, a focus on the ways in which experiences of prison are inscribed on the bodies of those who pass through the prison system (Moran, 2012). Furthermore, Norwegian research has critically examined aspects of Goffman's theory to interrogate concepts of ‘inside and ‘outside’ in relation to prison settings (Baer & Ravneberg, 2008). Changes in the ways that prisons are connected, through, for example, facilitating access to educational opportunities (Pike & Adams, 2012) in ways that were inconceivable in 1961 when Goffman published Asylums. This includes accessing Open University courses (Earle & Mehigan, 2019), in which some of the participants in this study were participating in. Changes and increasing levels of visitation also have consequences for the prison as a total institution (McCarthy & Adams, 2017). Additionally, there has been a significant increase in outside agencies, including the voluntary sector (Abrams et al., 2016), sports organisations (Meek, 2014) and other agencies working and delivering community services within prisons, as well as prison officers working outside of the prison within the community (|Maycock et al., 2020a, 2020b). Cumulatively this literature suggests a blurring of the boundaries between custody and community.

Recent developments in communication, in particular, the increasing use of video links between prisons and courts and to facilitate virtual visits with family and friends are further increasing the permeability of the prison walls (McKay, 2016). This includes both official and unofficial forms of communication, representing another insight into the ways in which the prison walls were becoming increasingly permeable. Research has indicated that the frequent absence of computers and internet access within prisons constitutes a particular manifestation of wider digital divides (Reisdorf & Jewkes, 2016). This is a continuation of the prevention of communication that has been part of prison life since Victorian times (Jewkes & Johnston, 2009), such prevention took on a heightened significance during the COVID-19 pandemic and lockdown (Blomberg et al., 2021).

The research cited here illustrates that despite its regular application to prisons, Goffman's theory of the total institution remains contested and debated within penology, in particular, due to the impacts of technology on connectedness between those in custody and the community. To summarise the critiques in this section, these tend to question the applicability of the total institution as a theory to analyse contemporary prisons, given the diverse ways that contemporary prisons are increasingly porous in ways that Goffman did not and could not have considered.

## The Scottish penal context

Within the distinct and devolved Scottish penal context within which this research is located (Croall et al., 2016; McNeill, 2016; Morrison, 2016; Morrison, 2012; Sparks & Morrison, 2015), the 2013 SPS organisational review (SPS, 2013), remains a formative influence on penal policy. This organisational review has desistance as its theoretical underpinning (McNeill, 2016), and more widely it is within the SPS that desistance theories have had a particular impact, with desistance theory subsequently reflected in a number of policy documents (SPS, 2016). This is a policy context emphasising the ‘assets’ people bring with them into the prison. An article from some senior SPS staff (including the then SPS Chief Executive) illustrates the ways in which assets were conceptualised often relating to connections outside of prison, within a wider policy context where desistance theories were formative:

…our vision must be one that encompasses the strengths and potential found within an individual as well as drawing on the assets found around them within families, their social networks and their communities. (McConnell et al., 2013, p. 11)

Reflecting this focus on connections outside of prison at an individual prisoner level, alongside the influence of desistance, there emerges a new emphasis within Scottish penal policy following the 2013 organisational review. This new direction has resulted in closer links and connections between custody and community across a range of policy areas, resonating with the critiques of the total institution outlined previously, in as much as these policies increased the ‘porousness’ of the prison walls across Scotland.

### ‘Equivalence’ through progressive penal policy reform in Scotland from 1993 to 2022

A wide range of literature has explored diverse aspects of equivalence between services within custody and community settings (Exworthy et al., 2012), often focusing on health services (Forrester et al., 2013; Levy, 1997; Lines, 2006; Niveau, 2007). To a significant extent, the shifts identified in the decline in relevance of the total institution are reflected in recent penal policymaking implemented in Scotland following the establishment of the SPS in 1993 and further shaped by the 2013 organisational review. Cumulatively, this might be summarised as ‘progressive penal transformation’ (Brangan, 2019), embodied in the following specific SPS policies and shifts in prison practice:

- Prison healthcare – the responsibility for provision of healthcare to prisoners in Scotland was transferred from the SPS to the NHS in 2011, with a focus on the continuity of healthcare provision, before, during and after prison.
- SPS Gender Identity Policy (2014) – this policy emerges out of the 2010 Equality Act.
- SPS Family strategy (2017) focused on improving outcomes for people in custody and their families.
- SPS and Third Sector Strategic Engagement (2017) sought to outline a strategic approach for the SPS to work with the third sector
- SPS Strategy Framework for the Management of Women Offenders in Custody (2017) – this framework aims to take account of the particular needs of women in custody. This includes housing some women in custody in new Community Custody Units (CCUs) resulting in some women in custody being closer to communities when these open in 2022.
- Vision for young people in custody (2014 and 2021) aims to prepare young people for a positive future, and takes account of the intention to integrate into Scottish law the UN Convention on the Rights of the Child.

Across these policies, visions and strategies one of the central underlying principles is the desirability of equivalence between the services provided within custody and the community. There is an assumption across these documents that access to community services and networks (such as health, education and family) and increasing proximity to community services and resources are positive for people in custody. For example, the two new CCUs for women in custody in Scotland are predicated on the idea that women in custody being closer to communities in more open community-focused units will have positive implications for rehabilitation. While the implications of such shifts in policy for already stretched community services have not yet been analysed, cumulatively these policies sidestep the larger questions about the size and reach of the criminal justice system in Scotland (McNeill, 2018).

### The COVID-19 lockdown and associated policy in Scotland

While the literature on the impacts of COVID-19 in prison settings is emerging (Gray et al., 2021; Rapisarda & Byrne, 2020), there is relatively little published specifically relating to Scotland (Armstrong et al., 2022; Maycock, 2021a; Maycock & Dickson, 2021; Morrison & Graham, 2022). Similarly, to many other jurisdictions in response to the COVID-19 pandemic, Scottish prisons went into lockdown in March 2020. At the time of submission in December 2022, aspects of the lockdown have been eased and reintroduced since March, however, elements of the lockdown introduced in March 2020 have been large, although not completely removed. Within Scotland, there have been some reports of both cases and deaths due to COVID-19 amongst both staff and inmates and outbreaks in particular prisons. In relation to everyday impacts of the COVID-19 pandemic, the lockdown in Scottish prisons has resulted in many aspects of prison life being paused, such as prison gyms and education departments, multi-faith centres being closed and family visits stopped. Subsequently virtual visits have been taking place, and family visits were restarted on 26 April 2021, but these were subject to change and disruption due to local COVID-19 outbreaks when they occurred.

The policy response to the COVID-19 pandemic by the SPS and Scottish Government in many senses feels quite different from the above policy and strategy. In contrast to the previous policy that had an unpinning principle of enhanced equivalence between services in custody and the community, the policy developed in response to COVID-19 has resulted in significant increases in in-cell time and less contact between people in custody and their families, out-of-cell activities and accessing services within the prison and various outside agencies. Moving in a contrasting direction to the policy and strategy outlined above, a significant change to the Prison Rules was implemented in April 2020, in response to the COVID-19 pandemic, through the following amendment: Scottish Parliament: The Prisons and Young Offenders Institutions (Scotland) Amendment Rules 2020: http://www.legislation.gov.uk/ssi/2020/122/article/2/made. This amendment to the prison rules provided new powers for the early release of a specific class of prisoners held in Scottish prisons. It also gave governors new powers, including the ability to suspend work, education and recreation, as well as changing access to showers from daily to at least twice weekly. Cumulatively, these changes caused some concern in relation to the human rights implications of these changes, expressed by a number of organisations including Her Majesty's Chief Inspector of Prisons for Scotland (HMIPS), the Scottish Human Rights Commission and the Scottish Prisoner Advocacy & Research Collective (SPARC). Within the context of the *formally administered* aspects of the total institution Goffman highlighted, this article is concerned with the implications of what these policy changes mean for the extent to which prisons have become increasingly *closed off* or *closer to* the communities around them.

## Methods and ethics

Following approval by the SPS Research Access and Ethics Committee, this project used a participatory correspondence methodology using the postal service in order to engage with a group of people in custody. Of the 13 participants invited to take part in the study, 8 gave their informed consent after having been sent the project information sheet and consent form in the post. The participants were all male, serving a long-term sentence, identified as white and were living in single-cell accommodation. Eight participants equate to a 0.1% sample of the entire Scottish prison population (official figures indicate that the average prison population in Scotland was around 8200 in 2019–2020 (Scottish Government, 2020b)). Consequently, this study makes no claims to be able to account for or to represent the many diverse experiences of lockdown across the Scottish prison estate, not least as the particular experiences of young people, women in custody and those on remand are not included in this study. It is hoped that this article illuminates and sensitively analyses the views of the eight men who chose to participate. This sample was pragmatic as the SPS ethics committee gave the approval to correspond to potential participants in the previous PAR study, and no one else either living or working in Scottish prisons during the COVID-19 pandemic.

Within prison settings, correspondence methods have been very rarely used (Brown, 2014; Ford & Berg, 2018; Walker et al., 2017), as the normative approach to prison research is through face-to-face methodologies with telephone or email methods used even more infrequently. Within the research context shaped by the COVID-19 pandemic, correspondence methods were the only means through which it was possible to engage with a group of people in custody in order for them to share their views on the COVID-19 pandemic in prison. Other aspects of this study are reported elsewhere (|Maycock, 2021a, 2021b; Maycock & Dickson, 2021), it is hoped that this method will be used in other studies with hard to reach research participants who are not able to participate in face to face data collection.

Despite most services (such as education, prison gyms and prison programmes), being paused, post was delivered as normal through the pandemic, so using the postal system was the only means of undertaking research. This study enabled participants to shape the research questions that formed the focus of each of the letters. This resulted in at times difficult and unexpected questions to be asked throughout the letters that constitute the data analysed in this article. Some of these questions related to the ways in which the COVID-19 pandemic deepened the experience of prison, and for the participants in this study made ‘outside world’ seem further away and more detached than it ever had. Through engaging with the participants in the first instance and their suggested questions shaping subsequent correspondence, it is hoped that responses to the second letter are able to shape wider research agendas on COVID-19 within prison settings.

The first letter was sent in April 2020 and the last in September 2021, therefore, this study relates to this particular period and the impacts of COVID-19 within this time frame, and does not speak to what happened outside of these dates. Consequently, it is important to state that this study does not analyse the implications of the easing on lockdown conditions outside of this timeframe. Subsequent to the last letter in September 2021, there has been a continued erosion of lockdown conditions within Scottish prisons. In February 2023, some concerns remain about aspects of Covid-19 restrictions being in place in Scottish Prisons.^3^ However, life in prison has in the main returned to the flows of prison life evident prior to the pandemic, although some more peripheral elements of the measures introduced during the COVID-19 remained in December 2022. Participants suggested that this mainly related to a sustained change in the reception process on entry to prison, and continued long hours spent within cells (which is potentially a consequence of staffing shortages and not a legacy of COVID-19 restrictions).

Each of the eight participants has been given pseudonyms to protect their identity with the prison referred to as HMP A. While there are recurring concerns about the literacy levels of people in custody (Creese, 2016; Morgan & Kett, 2002; Vacca, 2004), given that the researcher for this study knew all the participants from a previous project, these issues were relatively minimal given the relatively high levels of literacy amongst this group of participants. This suggests that this sample in some senses is relatively unusual, which in this instance might be a function of participants in the previous study engaging well with the prison education department with some participants quite advanced in an Open University degree. At all times, letters were written in plain English to further enhance the accessibility of the study.

All letters were sent in a stamped addressed envelope with the name and workplace address of the researcher, to enable all participants to respond to any letters without having to pay themselves for an envelope or stamp. All participants were encouraged to write at any time about their experiences of the pandemic and two did this not in response to a letter from the author. In total, 20 letters were received constituting a 30% response rate. Two drawings were also received. All responses were entered into a spreadsheet and then analysed in Nvivo 12 using an inductive thematic analysis (Bazeley & Jackson, 2013). Analysis of the letters sought to identify and consolidate themes emerging across the letters and to make connections with relevant literature. Themes and aspects of this study not analysed in this article are reported elsewhere (|Maycock, 2021a, 2021b; Maycock & Dickson, 2021).

## Findings

Findings are organised around two main areas: aspects of the data that suggest that participants were increasingly cut-off or conversely increasingly close to both everyday life within the prison and outside of the prison walls. However, it is important to note that at times, both these processes were evident complicating the analysis of the data in relation to Goffman's theory of the total institution as at times aspects of the data simultaneously illustrates both a cutting off and an increased closeness.

### Increasingly ‘cut-off’ – the re-emergence of the prison as a total institution during the COVID-19 pandemic

A significant proportion of the narratives included in the letters received in this study explored feelings of isolation and detachment both from everyday life within the prison and from families and communities more widely. For some participants, not having family visits was particularly challenging:

No family visits. I am missing having a reason to get out of bed. (Nigel)

The lack of family visits deepened feelings of isolation and detachment from life both within and outside the prison walls. For James, being detached from family related to how being in prison lockdown meant that he couldn’t protect and support his family in the context of the lockdown:

Personally, I am incredibly worried about progression so I can be back with my family supporting them at such hard times.

The collapse of the provider role that some men play within their families is a recurring concern for some men in prison (Arditti et al., 2005, Hairston, 2002, Dyer, 2005). Here we see the COVID-19 pandemic further undermining these aspects of male roles in some families. For James in particular, the COVID-19 pandemic and associated lockdown were having profound implications for his family relationships. These implications were deepened by what James viewed as poor management of the pandemic within custody. Below James reflects on the reasons why he didn’t want to use the phone in prison as much as he was able, as he felt that it wasn’t cleaned enough, therefore, increasing the chance of infection spreading:

Due to lack of cleaning of the phones in the sections and no hand sanitiser around. Once/Twice per month a call to one person in case I catch the virus. (James)

This was particularly significant, given that the telephone was the main means of staying in touch with family for the participants, while in-person visits were cancelled. That James felt the phone wasn’t cleaned enough points to a lack of faith in the cleaning routine and management of the pandemic in this regard. These apprehensions relating to the risk of infection within the prison were challenging for the participants, given that their options for getting out of what they felt were risky situations were limited. Additionally, prison staff potentially bringing COVID-19 into the prison was the greatest threat that they faced. In some senses within the quote above, we see both a cutting off and further distance from family, as well as a fear of being closer to the threat of the virus.

As well as these challenging increasing distance between the participants in this study and their families, there were profound changes to the prison regime as a consequence of the COVID-19 lockdown in prison that created a new separation between people in custody (Maycock, 2021a). This disrupted the everyday routines and rhythms in prison, so alongside the disruption to family visits explored above, there was disruption caused to the following services; library, prison programmes, most work sheds, education and gyms were all closed or cancelled. Participants reported being locked up in their cells for 23 h a day. John in particular found these changes hard and wanted a return to the pre-lockdown regime as soon as possible:

No education, no visits, no library. Locked up 23 h, no access to friends as hall locked up each side. Quite lonely, isolated.

Here we see John reflecting that due to being locked up for so much time, there was a further feeling of being cut off from friends within the prison, such sentiments of isolation echo more personal forms of crisis experienced during times of segregation. There was a recurring sense that while this was a high-security prison, the pre-lockdown life was missed, and the pre-lockdown regime and associated routine was something that a number of participants wanted to get back to. There was also a sense in some of the letters that due to the nature of the lockdown in prison, the ways in which cells were seen were being reconsidered. Importantly, Peter below reflects on the ways he viewed his cell in a new way, newly framed as more of a cage than a cell:

Eating/Sleeping/toilet all in your cell – very cage like.

The significance of this quote relates to Peter being a long-term prisoner who had spent many years in prison so had become quite used to his cell prior to the pandemic, and the lockdown resulted in him reconsidering this space. Extended periods within their cells fostered feelings of boredom, frustration and stress amongst the participants in this study, feelings more often reported by prisoners in segregation units of prisons (Brown, 2020). This section has identified people in custody feeling increasingly cut off from both wider connections to family and friends outside of prison as well as friends within the prison. The following section explores counter processes and experiences that to an extent brought the participants in this study closer to life outside of prison.

### Increasingly ‘connected’ – he subversion of the prison as a total institution during the COVID-19 pandemic

Resonating with some of the well-developed critiques of Goffman's theory of the total institution explored above, there were also parts of the letters where participants indicated they felt in some ways closer to life outside of prison. This section explores the aspects of the letters that question or complicate the section above in as much as it points to aspects of the COVID-19 pandemic and lockdown that participants felt brought everyday life in prison closer to that of everyday life outside of prison. Alongside and intertwined with the reflections above, there emerges counter-narratives or reflections and processes that brought participants closer to various aspects of the COVID-19 pandemic. Shifts in SPS policy (or changes in the formal administration of prison life to return to Goffman) such as the introduction of virtual visits or mobile phones that had important implications for the connections of people in custody to their family and friends in the community. This points to a complication of the porousness and re-emergence of the prison as a total institution and to an extent brings into question the relevance of this theory within the particular context of the COVID-19 pandemic. This section explores changes in policy and practice as well as experiences that might be seen to dissolve aspects of the prison walls, by bringing people in custody closer to the COVID-19 virus, vaccines, families, friends and wider community on the other side of the prison walls.

Perhaps the most obvious aspect of feeling closer to life and events beyond the prison walls is related to the perception of threat and/or fear of the COVID-19 virus being imported into prison from the community. At the time of data collection (April–September 2021) there had been no outbreaks of COVID-19 in this prison, so the participants in this study felt that the COVID-19 virus could only be imported into the prison from outside, most likely by a member of prison staff. As a consequence of this new perception of threat, prison staff was viewed differently than they had been previously, as they constituted a new type of threat relating to transmitting the virus. A number of participants reflected that they were concerned about a lack of social distancing in prison, which proved to be consistently challenging within a prison not designed with this in mind. Additionally, due to high and changing levels of staff sickness, Nigel below indicates that he felt that the high levels of staff turnover further increased the risk that staff represented to people in custody:

No social distancing that equals to what's forced on prisoners. Personally, I feel the staff changes on landings is too often bringing more risk of infection as no one wears a mask or gloves when in contact with prisoners.

This quote from Nigel provides a new insight into the ways that people in custody viewed prison staff during the pandemic, similar sentiments was shared by the majority of participants. This provides an insight into one aspect of the ways in which the COVID-19 pandemic influenced or disrupted what Liebling and Arnold call ‘order’ in prison (Liebling & Arnold, 2004):

“…the degree to which the prison environment is structured, stable, predictable and acceptable.” (Liebling & Arnold, 2004, p. 291)

The quote above also provides insights into the ways that Nigel viewed risks associated with COVID-19 transmission in the prison. It also illustrates the links between the prison and the wider community from which prison staff came to the prison from. Goffman explores staff/prisoner interactions in total institutions in a number of instances:

“Just as neither the staff nor the inmate group is homogenous so a simple division between staff and inmate groups can sometimes conceal important facts.” (1961, p. 109)

There has been some subsequent critique of Goffman's analysis of staff/prisoner interactions indicating that they are not always so fixed and/or necessarily hostile (Ben-David, 1992). In the quote above and further reflections from Nigel below, we get a new insight into these relationships within prisons. It is evident that the COVID-19 pandemic was having implications not only for how the participants in this study viewed their time in prison, the physical space of their cells etc., but also how they viewed the prison staff that they interacted with. Nigel went further, and was evidently quite concerned about staff transmitting the virus into the prison, as he was unsure if prison staff were being tested:

When staff enters the prison are they being tested?

The lack of COVID-19 cases within the prison at the time of many of the letters were received, caused some frustration for a number of participants, given that restrictions in prison didn’t seem to take account of this:

Why can't prisoners go to the gym; there has been no confirmed cases of Covid-19 within HMP A Why are prisoners in HMP A locked up 22 h a day when there has been no confirmed cases of Covid-19. (James)

These frustrations associated with being locked down and having their normal routine disrupted due to COVID-19, combined with the feeling that the threat of the virus was being imported into the prison by prison staff in important ways brought the participants in this study closer to life outside of the prison walls. These aspects of the letters constitute a challenge to the notion that prisons are ‘cut-off’ in simplistic ways from life, events and threats that happen outside of the prison.

A further important subversion of the ways in which prisons were connected to life outside of the prison relates to the availability of COVID-19 vaccines and testing within this prison. The availability of COVID-19 testing and vaccinations in prison represents the other significant connection between prison and life outside of the prison walls. There is some emerging evidence of vaccination hesitancy within prison settings (Geana et al., 2021; Khorasani et al., 2021) and emerging evidence of the complexities of COVID-19 testing within prisons (Lemasters et al., 2020). A further letter was sent to all eight participants in October 2022, in order to better understand the experiences of COVID-19 vaccination amongst the participants, there was one response to this letter. In this letter, Nigel reflected on the continuing aspects of the lockdown in this prison, some 19 months after prisons in Scotland initially went into lockdown. He also indicated that he was isolating due to having a positive COVID-19 test result:

[As a consequence of a positive Covid test result] At the moment I’m locked up 23 h a day! I only get one hour exercise at 8am. Lockdown at the moment is like being in the segregation unit.

In this letter, Nigel indicates that he has had both vaccines, and got them at the earliest opportunity. Importantly, it is unclear what the introduction of video visits, or the provision of mobile phones to people in custody have meant, as these changes have not been analysed, but it is expected that they will have enabled people in custody to connect to friends and family in ways not possible before the pandemic. These changes constitute important further aspects of increased connectedness during the COVID-19 pandemic. Mobile phones have been handed out to all prisoners in Scotland, which is a major change in terms of people in custody being to stay in touch with their families and friends. However, at the time of data collection, it was not possible to include this in the analysis here. Virtual visits have been rolled out across the prison estate which John felt to be a positive development that has happened specifically during the time of the pandemic, as John reflections below:

I have had several virtual visits. I think they are excellent, especially for people like myself who have children who do not know the true location of their fathers. (John)

More research is required on the impacts of both mobile phones and virtual visits, and how people in custody experience these changes and what this means for the connections between people in custody and the community? Questions also remain about the longevity of these changes after the COVID-19 pandemic has less influence in custodial as well as community settings. This section has illustrated many of the ways in which participants felt closer and more connected in ways that are specific to the COVID-19 pandemic. It is evident from this section that prison walls in Scotland are quite permeable to the COVID-19 virus, vaccinations and new technologies.

## Conclusion – the re-emergence of the prison as a total institution during times of crisis

The findings within this study complicate pre-pandemic literature that has engaged with Goffman's theory of the total institution, which has suggested a gradual erosion of the relevance of Goffman's notion of the prison as a total institution, and in some senses the softening and increasing permeability of the prison walls. While the letters analysed in this article to an extended support these pre-pandemic critiques of Goffman's theory in relation to prisons, the prison policy and practice shifts made in response to the COVID-19 pandemic also complicate the extent to which the theory of the total institution remains a useful theoretical lens with which to analyse prisons within the COVID-19 and post-COVID-19 contexts.

The time of crisis that the COVID-19 pandemic represents within the Scottish prison system resulted in the participants in this study feeling increasingly cut off from everyday life both within prison and outside of the prison walls. There was a recurring sense in the letters that the COVID-19 lockdown felt like an additional punishment, not unlike segregation (another manifestation of crisis, although at a more individual level). This can be seen as a ‘deepening’ of time in prison during the COVID-19 lockdown, with participants in this study feeling increasingly cut off from the ‘outside’ world. Reflecting on the letters received as part of this project, it is difficult to conceive of the COVID-19 pandemic as anything other than transformative for everyday prison life, and this was causing the participants in this study significantly enhanced pains and frustrations associated with imprisonment (Sykes, 1958).

Conversely, alongside and intertwined with these aspects of the letters analysed in this study, there were also some instances of increased closeness to the world beyond the prison walls, for example, through fears relating to the COVID-19 virus getting into the prison and the availability of COVID-19 vaccines in prison. Additionally, other changes in response to COVID-19 (such as the introduction of mobile phones and virtual visits) that were implemented to enable connections between people in custody and their families, further increased the closeness of the outside world. Such changes require further analysis, as it is expected that both these aspects of innovation will have important implications for the ways that people in custody could connect to the outside world, although connections to friends and staff within prison remained complicated.

Methodologically, this study used letter writing as a means of reaching and engaging with a group of prisoners, who as a consequence of the COVID-19 pandemic were not able to participate in this study in conventional pre-pandemic ways. Correspondence methods have significant potential to enable other research participants that might be hard to reach or in some way cut off from in person forms of data collection for a range of reasons. The analysis in this article raises fundamental questions about the nature of contemporary imprisonment in the COVID-19 era, and the extent to which Goffman's theory of the total institution still has utility as a theory to analyse contemporary prisons. The narratives analysed in this article point to a more essential (and total) nature of contemporary imprisonment, that is clearest and most evident at times of crisis, but for various reasons (such as the policies, strategies and research explored above) are less evident due to progressively closer connections between prisons and wider communities over recent years. Therefore, it will be important to continue to explore the ways in which the COVID-19 pandemic and the crisis response to the pandemic by prison managers and administrators, have led to a significantly different direction in penal policymaking and practice.

As has been illustrated in this article, the unprecedented COVID-19 times have resulted in complicated and often contradictory changes within Scottish prisons. This was a time of crisis experienced by both prison staff, managers and people in custody with further longitudinal research required to analyse the ongoing legacy of this particular crisis within prison systems. More widely this article suggests that times of crisis in prison, at a range of levels such as personal (segregation), prison specific (riots) or prison system wide (response to COVID-19 and other pandemics) are times during which prisons are experienced as an *increasingly* total institution. The response to the COVID-19 pandemic suggests that in response to a crisis such as the COVID-19 pandemic, prison authorities are able both enhance and decrease the total nature of prisons depending on the threat faced. Ultimately, it is during times of crisis (previous, current and future), that it is possible to see the possibilities of the more total aspects of prison settings being realised, this in turn ensures that Goffman's theory of the total institution remains a useful lens through which to analyse contemporary experiences of imprisonment.

## Notes

1. In Scotland, this equates to a sentence of over four years.

2. For example, the then Scottish minister for community safety, Ash Denham reflected on two prison visits in September 2021 ‘…that's something not just evident at these two prisons, but across the whole of the Scottish prison estate, where the positivity and response of staff and co-operation by those in care has ensured stability during an incredibly challenging time.’ https://www.scotsman.com/news/politics/how-scotlands-prison-service-is-coping-with-emerging-from-covid-ash-denham-3370328 (accessed, 28 September 2021).

3. Concerns remain about Covid-19 restrictions in Scottish prisons still being place in February 2023, as illustrated by the following a Scottish Parliamentary Question tabled on 23 February 2023 by Pauline McNeill (MSP): To ask the Scottish Government what recent discussions it has had with the Scottish Prison Service regarding the lifting of all Covid-19-related restrictions in prisons. (S6O-01927).

## References

[bibr1-26338076231165116] Abrams L. S. Hughes E. Inderbitzin M. Meek R. (2016). The Voluntary Sector in Prisons. Encouraging Personal and Institutional Change. London: Palgrave.

[bibr2-26338076231165116] Adams R. (2016). Prison Riots in Britain and the USA. Springer.

[bibr73-26338076231165116] Arditti J. A. Smock S. A. Parkman T. S. (2005). “It's been hard to be a father”: A qualitative exploration of incarcerated fatherhood. Fathering: A Journal of Theory, Research & Practice about Men as Fathers, 3(3), 267–288. 10.3149/fth.0303.267

[bibr3-26338076231165116] Armstrong S. Barkas B. Casey R. Cornish N. Gormley C. McNeill F. Schinkel M. (2022). *Prisoner experiences of Covid-19 restrictions in Scotland during 2020*. Glasgow: University of Glasgow Working Paper.

[bibr4-26338076231165116] Baer L. D. Ravneberg B. (2008). The outside and inside in Norwegian and English prisons. Geografiska Annaler: Series B, Human Geography, 90(2), 205–216. 10.1111/j.1468-0467.2008.00287.x

[bibr5-26338076231165116] Baumer E. P. O'Donnell I. Hughes N. (2009). The porous prison. The Prison Journal, 89(1), 119–126. 10.1177/0032885508330430

[bibr6-26338076231165116] Bazeley P. Jackson K. (2013). Qualitative data analysis with NVivo. Sage Publications Limited.

[bibr7-26338076231165116] Ben-David S. (1992). Staff-to-Inmates relations in a total institution: A model of five modes of association. International Journal of Offender Therapy and Comparative Criminology, 36(3), 209–219. 10.1177/03066249203600305

[bibr8-26338076231165116] Blomberg M. Altschwager D. Seo H. Booton E. Nwachukwu M. (2021). Digital divide and marginalized women during COVID-19: A study of women recently released from jail. Information, Communication & Society, 24(14), 2113–2132. 10.1080/1369118X.2021.1963462

[bibr9-26338076231165116] Brangan L. (2019). Civilizing imprisonment: The limits of Scottish penal exceptionalism. The British Journal of Criminology, 59(4), 780–799. 10.1093/bjc/azy057

[bibr10-26338076231165116] Brown E. (2020). A systematic review of the effects of prison segregation. Aggression and Violent Behavior, 52, 101389. 10.1016/j.avb.2020.101389

[bibr11-26338076231165116] Brown G. R. (2014). Qualitative analysis of transgender Inmates’ correspondence:Implications for departments of correction. Journal of Correctional Health Care, 20(4), 334–342. 10.1177/1078345814541533 25038142

[bibr12-26338076231165116] Carrabine E. (2005). Prison riots, social order and the problem of legitimacy. British Journal of Criminology, 45(6), 896–913. 10.1093/bjc/azi052

[bibr13-26338076231165116] Colvin M. (1992). The penitentiary in crisis: From accommodation to riot in New Mexico. SUNY Press.

[bibr14-26338076231165116] Comfort M. (2009). Doing time together: Love and family in the shadow of the prison. University of Chicago Press.

[bibr15-26338076231165116] Cox G. H. Rhodes S. L. (1990). Managing overcrowding: Corrections administrators and the prison crisis. Criminal Justice Policy Review, 4(2), 115–143. 10.1177/0887403490004002

[bibr16-26338076231165116] Creese B. (2016). An assessment of the English and maths skills levels of prisoners in England. London Review of Education, 14(3), 13–30. 10.18546/LRE.14.3.02

[bibr17-26338076231165116] Croall H. Mooney G. Munro M. (2016). Crime, justice and society in Scotland. London: Routledge.

[bibr18-26338076231165116] Davies C. (1989). Goffman's concept of the total institution: Criticisms and revisions. Human Studies, 12(1), 77–95. 10.1007/BF00142840

[bibr74-26338076231165116] Dyer W. M. (2005). Prison, fathers, and identity: A theory of how incarceration affects men's paternal identity. Fathering: A Journal of Theory, Research & Practice about Men as Fathers, 3(3).

[bibr19-26338076231165116] Earle R. Mehigan J. (2019). Degrees of Freedom: Prison Education at The Open University. Policy Press.

[bibr20-26338076231165116] Ellis R. (2021). Prisons as porous institutions. Theory and Society, 50(2), 175–199. 10.1007/s11186-020-09426-w

[bibr21-26338076231165116] Evans P. (1980). Prison crisis. Allen & Unwin.

[bibr22-26338076231165116] Exworthy T. Samele C. Urquía N. Forrester A. (2012). Asserting prisoners’ right to health: Progressing beyond equivalence. Psychiatric Services, 63(3), 270–275. 10.1176/appi.ps.201100256 22388532

[bibr23-26338076231165116] Farrington K. (1992). The modern prison as total institution? Public perception versus objective reality. NPPA Journal, 38(1), 6–26. 10.1177/001112879203800100

[bibr24-26338076231165116] Fitzgerald M. Sim J. (1980). Legitimating the prison crisis: A critical review of the may report. The Howard Journal of Criminal Justice, 19(1-3), 73–84.

[bibr25-26338076231165116] Ford L. T. Berg J. D. (2018). Analytical evidence to show letters impregnated with novel psychoactive substances are a means of getting drugs to inmates within the UK prison service. Annals of Clinical Biochemistry: International Journal of Laboratory Medicine, 55(6), 673–678. 10.1177/0004563218767462 29534614

[bibr26-26338076231165116] Forrester A. Exworthy T. Olumoroti O. Sessay M. Parrott J. Spencer S.-J. Whyte S. (2013). Variations in prison mental health services in England and Wales. International Journal of Law and Psychiatry, 36(3-4), 326–332. https://doi.org/10.1016j.ijlp.2013.04.007 23669592 10.1016/j.ijlp.2013.04.007

[bibr27-26338076231165116] Geana M. V. Anderson S. Ramaswamy M. (2021). COVID-19 vaccine hesitancy among women leaving jails: A qualitative study. Public Health Nursing, 38(5), 892–896. 10.1111/phn.12922 33973268 PMC8242643

[bibr28-26338076231165116] Goffman E. (1961). Asylums : Essays on the social situation of mental patients and other inmates (1st ed.). Anchor Books.

[bibr29-26338076231165116] Gray R. Rooney B. Connolly C. (2021). Experiences of COVID-19 isolation in Northern Ireland prisons: a qualitative study. International journal of prisoner health, 17(3), 304–319. 10.1108/IJPH-09-2020-0076

[bibr75-26338076231165116] Hairston C. F. (2002). Fathers in prison: Responsible fatherhood and responsible public policies. Marriage & Family Review, 32(3-4), 111–135.

[bibr30-26338076231165116] Jewkes Y. Johnston H. (2009). ‘Cavemen in an era of speed-of-light technology’: Historical and contemporary perspectives on communication within prisons. The Howard Journal of Criminal Justice, 48(2), 132–143. 10.1111/j.1468-2311.2009.00559.x

[bibr31-26338076231165116] Khorasani S. B. Koutoujian P. J. Zubiago J. Guardado R. Siddiqi K. Wurcel A. G. (2021). COVID-19 Vaccine interest among corrections officers and people who are incarcerated at middlesex county jail, massachusetts. Journal of Urban Health, 98(4): 459–463. https://doi.org/10.1007/s11524-021-00545-y 34041670 10.1007/s11524-021-00545-y PMC8152706

[bibr32-26338076231165116] Lemasters K. McCauley E. Nowotny K. Brinkley-Rubinstein L. (2020). COVID-19 cases and testing in 53 prison systems. Health & Justice, 8(1), 1–6. 10.1186/s40352-020-00125-3 33306151 PMC7729147

[bibr33-26338076231165116] Levy M. (1997). Prison health services: Should be as good as those for the general community. British Medical Journal Publishing Group. 10.1136/bmj.315.7120.1394

[bibr34-26338076231165116] Liebling A. Arnold H. (2004). Prisons and Their Moral Performance: A Study of Values, Quality, and Prison Life. Oxford University Press.

[bibr35-26338076231165116] Lines R. (2006). From equivalence of standards to equivalence of objectives: The entitlement of prisoners to health care standards higher than those outside prisons. International journal of prisoner health, 2(4), 269–280. 10.1080/17449200601069676.

[bibr36-26338076231165116] Losel F. (2007). The prison overcrowding crisis and some constructive perspectives for crime policy. The Howard Journal of Criminal Justice, 46, 512–519. 10.1111/j.1468-2311.2007.00497.x

[bibr37-26338076231165116] Mac Suibhne S. (2011). Erving Goffman’s Asylums 50 years on. British Journal of Psychiatry, 198(1), 1–2. 10.1192/bjp.bp.109.077172. 21200067

[bibr38-26338076231165116] Maycock M. (2021a). ‘COVID-19 has caused a dramatic change to prison life’. Analysing the impacts of the COVID-19 pandemic on the pains of imprisonment in the Scottish prison estate. The British Journal of Criminology, 62(1), 218–233. 10.1093/bjc/azab031

[bibr39-26338076231165116] Maycock M. (2021b). “I do not appear to have had previous letters.” the potential and pitfalls of using an action research correspondence method to facilitate insights into life in prison during the COVID-19 pandemic. International Journal of Qualitative Methods, 20, 1–11. 10.1177/16094069211047129

[bibr40-26338076231165116] Maycock M. Dickson G. (2021). Analysing the views of people in custody about the management of the COVID-19 pandemic in the Scottish prison estate. International Journal of Prisoner Health, 17, 320–334. 10.1108/IJPH-09-2020-0065

[bibr41-26338076231165116] Maycock M. McGuckin K. Morrison K. (2020a). ‘We are “free range” prison officers’, the experiences of Scottish prison service throughcare support officers working in custody and the community. Probation Journal, 67(0), 358–374. 10.1177/0264550520954898

[bibr42-26338076231165116] Maycock M. McGuckin K. Morrison K. (2020b). What are the implications of changing place for the professional performativity of prison officers? Corrections, 57–73. 10.1080/23774657.2020.1821407

[bibr43-26338076231165116] McCarthy D. Adams M. (2017). Prison visitation as human ‘right’ or earned ‘privilege’? The differing tales of England/Wales, and Scotland. Journal of Social Welfare and Family Law, 39(4), 403–416. 10.1080/09649069.2017.1390292

[bibr44-26338076231165116] McConnell C. Carnie J. Mehta H. (2013). Reframing the role of custody within the desistance paradigm. Scottish Justice Matters, 1(2), 10–11.

[bibr45-26338076231165116] McKay C. (2016). Video links from prison: Permeability and the carceral world. International Journal for Crime, Justice and Social Democracy, 5(1), 21–37. 10.5204/ijcjsd.v5i1.283

[bibr46-26338076231165116] McNeill F. (2016). Desistance and criminal justice in Scotland. In Croall H. Mooney G. Munro M. (Eds.), Crime, justice and society in Scotland (pp. 14–235). Routledge.

[bibr47-26338076231165116] McNeill F. (2018). Pervasive punishment: Making sense of mass supervision. Emerald Group Publishing.

[bibr48-26338076231165116] Meek R. (2014). Sport in prison : exploring the role of physical activity in correctional settings. Routledge.

[bibr49-26338076231165116] Moran D. (2012). Prisoner reintegration and the stigma of prison time inscribed on the body. Punishment & Society, 14(5), 564–583. 10.1177/1462474512464008

[bibr50-26338076231165116] Moran D. (2013). Between outside and inside? Prison visiting rooms as liminal carceral spaces. GeoJournal, 78(2), 339–351. http://www.jstor.org/stable/42006323

[bibr51-26338076231165116] Moran D. (2014). Leaving behind the ‘total institution’? Teeth, transcarceral spaces and (re) inscription of the formerly incarcerated body. Gender, Place & Culture, 21(1), 35–51. 10.1080/0966369X.2012.759906

[bibr52-26338076231165116] Moran D. (2015). Carceral geography : spaces and practices of incarceration. Ashgate.

[bibr53-26338076231165116] Morgan M. Kett M. (2002). The prison adult literacy survey results and implications. Dublin: Irish Prison Service.

[bibr54-26338076231165116] Morrison K. (2016). The criminal justice system in Scotland. Criminology Oxford University Press.

[bibr55-26338076231165116] Morrison K. Graham H. (2022). Scotland. In Dünkel F. Harrendorf S. van Zyl Smit D. (Eds.), The impact of COVID-19 on prison conditions and penal policy. Routledge.

[bibr56-26338076231165116] Morrison K. (2012). *Penal transformation in post-devolution Scotland: Change and resistance*. [PhD thesis, University of Edinburgh], https://www.era.lib.ed.ac.uk/bitstream/1842/6435/2/Morrison2012.pdf

[bibr57-26338076231165116] Niveau G. (2007). Relevance and limits of the principle of “equivalence of care” in prison medicine. Journal of Medical Ethics, 33(10), 610–613. 10.1136/jme.2006.018077 17906061 PMC2652802

[bibr58-26338076231165116] Pike A. Adams A. (2012). Digital exclusion or learning exclusion? An ethnographic study of adult male distance learners in English prisons. Research in Learning Technology, 20(4). 10.3402/rlt.v20i0.18620

[bibr59-26338076231165116] Rapisarda S. S. Byrne J. M. (2020). The impact of COVID-19 outbreaks in the prisons, jails, and community corrections systems throughout Europe. Victims & Offenders, 15(7-8), 1105–1112. 10.1080/15564886.2020.1835768

[bibr60-26338076231165116] RCN (2016). *Five years on: Royal college of nursing scotland review of the transfer of prison health care from the scottish prison service to Nhs Scotland*. Edinburgh: Royal College of Nursing.

[bibr61-26338076231165116] Reisdorf B. C. Jewkes Y. (2016). (B)Locked sites: Cases of internet use in three British prisons. Information, Communication & Society, 19(6), 771–786. 10.1080/1369118X.2016.1153124

[bibr62-26338076231165116] Schliehe A. K. (2016). Re-Discovering goffman: Contemporary carceral geography, the “total” institution and notes on heterotopia. Geografiska Annaler: Series B, Human Geography, 98(1), 19–35. 10.1111/geob.12087

[bibr63-26338076231165116] Scottish Government (2020a). Coronavirus (COVID-19): Scotland's route map through and out of the crisis. Scottish Government.

[bibr64-26338076231165116] Scottish Government (2020b). *Scottish prison population: statistics* 2019 *to* 2020. Edinburgh: Scottish Government.

[bibr65-26338076231165116] Sessar K. (1994). Overcrowding-not the only crisis in the custodial system. European Journal on Criminal Policy and Research, 2, 107–116. 10.1007/BF02249444

[bibr66-26338076231165116] Shalev S. Edgar K. (2015). Deep custody: Segregation units and close supervision centres in England and Wales. London: Conquest Litho, 93, 14.

[bibr67-26338076231165116] Sparks R. Morrison K. (2015). Research, knowledge and criminal justice policy: The Scottish experience. In Crime, justice and society in Scotland (pp. 42–56). Routledge.

[bibr76-26338076231165116] SPS (2013). *Unlocking potential, transforming lives*. Edinburgh: Scottish Prison Service.

[bibr68-26338076231165116] SPS (2016). *Value proposition* Edinburgh: Scottish Prison Service.

[bibr69-26338076231165116] Sykes G. M. (1958). The Society of Captives : a Study of a Maximum Security Prison. Princeton University Press.

[bibr70-26338076231165116] Toch H. (2007). Men in crisis: Human breakdowns in prison. Transaction Publishers.

[bibr71-26338076231165116] Vacca J. S. (2004). Educated prisoners are less likely to return to prison. Journal of Correctional Education, 55(4), 297–305. http://www.jstor.org/stable/23292095

[bibr72-26338076231165116] Walker T. Shaw J. Turpin C. Roberts C. Reid C. Abel K. (2017). A qualitative study of good-bye letters in prison therapy. Crisis, 38(2), 100–106. 10.1027/0227-5910/a000411 27445012

